# A commentary on forging a path for CHANGE: culturally focused HIV training for the next generation in pursuit of equity

**DOI:** 10.1080/09581596.2024.2434472

**Published:** 2024-12-10

**Authors:** Jahn Jaramillo, Derrick Forney, Felicia O. Casanova, Naysha N. Shahid, Devina J. Boga, Nequiel Reyes, Renae Schmidt, Sannisha K. Dale, Daniel J. Feaster, Viviana E. Horigian

**Affiliations:** a Department of Public Health Sciences, University of Miami Miller School of Medicine, Miami, FL, USA; b Center for HIV and Research in Mental Health, University of Miami, Coral Gables, FL, USA; c Department of Psychology, University of Miami, Coral Gables, FL, USA

**Keywords:** Training programs, HIV, mental health, workforce, diversity

## Abstract

Training programs focused on developing the next generation of scholars with expertise in HIV and mental health are crucial for advancing health equity and cultivating a diverse workforce by supporting individuals with lived experience and a strong commitment to serving underserved communities. However, disparities persist in the workforce, particularly in the inclusion of professionals typically underrepresented in research. The aim of this commentary is to explore the strengths and challenges of a NIMH-funded training program (T32), Culturally focused HIV Advancements through the Next Generation for Equity (CHANGE), at the University of Miami, with the goal of providing a series of general recommendations. The program excels in leveraging Miami’s unique context, recruiting a cohort of trainees committed to addressing HIV and mental health inequities, delivering a tailored curriculum, and providing strong leadership and mentorship networks to trainees. Additional opportunities for training programs that attract minoritized scholars to realize their vision include further increasing underrepresented scholars in health research, expanding federal funding and institutional investment in training programs, continuing to combat systemic inequities, fostering culturally-sensitive mentorship training, and building upon existing resources to provide trauma-informed support that acknowledges and addresses the unique, intersectional, and historical trauma experienced by trainees. We close with calls to action spanning institutional, community, and policy levels, urging scientists and decision-makers to actively address disparities in diversifying the HIV workforce, fostering equity, and creating inclusive training environments.

## Background

Underserved communities of color are disproportionately impacted by the HIV epidemic in the United States (US) (Centers for Disease Control and Prevention, 2019, 2023; Patel et al., 2024). To end the HIV epidemic, effective strategies are needed to reach, care for, and overcome barriers that affect Black, Latine/x, and LGBTQIA+ communities (Pellowski et al., 2013). Due to socio-structural factors including racism, heterosexism, transphobia, and poverty, ending the HIV epidemic remains a distant goal for the most impacted communities, despite biomedical advances that offer effective treatment and prevention options (Bowleg et al., 2022; Cortes & Sued, 2024; Nosyk et al., 2020). In Miami, Florida, a key area of the US HIV epidemic, disparities are evident in new HIV diagnoses, with 29% among Black individuals, 59% among Latine/x individuals, and 68% among men who have sex with men (MSM) (Florida Department of Health, 2018). Engagement in care is suboptimal, with rates of 73% for Black individuals, 74% for Latine/x individuals, and 76% for MSM (Florida Department of Health, 2019a, 2019b, 2019c). Additionally, rates of viral suppression are low, at 64% for Black individuals, 67% for Latine/x individuals, and 66% for MSM (Florida Department of Health, 2019a, 2019b, 2019c).

Recent literature advocates for structural changes to systemic issues and their manifestations (e.g. limited access to care, housing) and an increased emphasis on tailored interventions for the most affected communities (Gilbert et al., 2021; Phillips et al., 2021; Vitsupakorn et al., 2023). Despite advances in research on cultural considerations for underserved communities in HIV prevention and care, there remains a significant gap between research findings and their translation into culturally relevant interventions (Guilamo-Ramos et al., 2023; Li et al., 2024; Rodriguez-Diaz et al., 2021). Although initiatives aimed at ending the HIV epidemic (EHE) support creative approaches to addressing HIV health disparities, there is a notable absence of culturally and racially appropriate interventions (Guilamo-Ramos et al., 2020; Vitsupakorn et al., 2023). This underscores the need for evidence-based interventions inclusive of Black, Latine/x, and LGBTQIA+ communities that are culturally tailored to address their unique needs and contexts (Bass et al., 2020; Cespedes et al., 2022; Dubé et al., 2022; Philbin et al., 2022; Watson et al., 2020). Moreover, community-engaged approaches offer a promising avenue for fostering collaborative partnerships and co-creation of interventions with communities to ensure interventions are culturally sensitive, linguistically appropriate, and accessible (Coleman et al., 2022; Fernandez et al., 2023; Gamarel et al., 2022). Relying solely on scientists is inadequate for developing effective interventions; meaningful progress must occur within the framework of community-engaged work, such as community-based participatory research (CBPR; Evans et al., 2023; Julian McFarlane et al., 2022).

Although diverse community organizations may be eager to collaborate (Robillard et al., 2022), researchers need to be trained in CBPR approaches to ensure bidirectional and mutually beneficial partnerships (i.e. the reciprocal exchange of knowledge and benefits between researchers and community partners), where both researchers and community members contribute expertise and benefit from the collaboration. CBPR training is also essential to prevent harm that can arise from historical (e.g. Tuskegee syphilis study, Havasupai Tribe Diabetes Project) and present-day exploitative or superficial forms of community engagement, such as when communities are treated as mere subjects of research rather than as equal partners (Brandt, 1978; Garrison, 2013). By fostering trust, respect, and shared decision-making, CBPR ensures that research outcomes align with community priorities and promote sustainable change (Breland-Noble et al., 2024; Morgan et al., 2021; Samuels et al., 2024).

Similarly, HIV disparities impacting key populations and the absence of culturally relevant interventions extend to both the healthcare workforce and research scientists working to address these gaps (Fitzpatrick et al., 2006; Fuchs et al., 2016; Irie et al., 2023; Sutton et al., 2021). Disparities in the HIV workforce persist, hindering progress toward ending the epidemic (Irie et al., 2023). For instance, the slow growth of diversity fellowships in National Institutes of Health (NIH) predoctoral programs, underscore the barriers faced by underrepresented individuals in the HIV research workforce (Hemming et al., 2019; Nguyen et al., 2022). Limited access to mentorship and research opportunities, financial constraints, and systemic biases in the selection process, impede the progress of investigators from underrepresented minority backgrounds (Edwards et al., 2022; Fitzpatrick et al., 2006; Webb, Guerau de Arellano, et al., 2022). Additionally, early-career scientists from historically marginalized groups face challenges like insufficient funding, limited institutional support, and a lack of diverse representation in leadership roles, hindering their crucial contribution to developing culturally tailored interventions for priority populations (Bonifacino et al., 2021; Fitzpatrick et al., 2006; Kreider et al., 2023; Peña et al., 2023). These individuals are often driven by an intrinsic motivation to pursue research on inequities that, while pervasive, are frequently overlooked and challenge the status quo within institutions resistant to change. Without targeted support, the weight of these obstacles can hinder their ability to thrive and make lasting contributions to their fields. These specific challenges contribute to the ongoing disparities in the HIV research workforce.

Promising approaches for addressing workforce challenges include training programs, such as the T32 Ruth L. Kirschstein Institutional National Research Service Award Program sponsored by the NIH (Valantine et al., 2016). These initiatives are awarded to universities to facilitate the recruitment and training of individuals in specified shortage areas, preparing predoctoral and postdoctoral students (hereafter referred to as ‘trainees’) for impactful careers addressing the nation’s health-related research needs, such as HIV (Sancheznieto et al., 2022). They actively cultivate needed expertise and may help shape a workforce with the necessary expertise (technical and lived) to serve marginalized communities. The growing focus on intersectionality, a framework rooted in Black feminism, unpacks how interlocking systems of oppression (e.g. racism, heterosexism) and privilege shape our experiences and navigation of the world (Collective, 1986; Crenshaw, 1989; Dale et al., 2022), has expanded to university training programs dedicated to developing future researchers (Torres Acosta et al., 2023). These programs aim to empower scholars to understand key factors and approaches as well as develop interventions for immediate impact and lasting progress (Irie et al., 2023). However, T32 programs vary in structure, objectives, and outcomes (Sancheznieto et al., 2022). Therefore, their continued development (Jackson et al., 2023) remains essential for tailoring training approaches, refining strategies, and optimizing their influence on the HIV and mental health workforce. Our T32 program, (Culturally focused HIV Advancements through the Next Generation for Equity: CHANGE henceforth) represents an urgent and necessary effort to integrate current understandings of inclusivity, intersectionality, and social justice within the traditional T32 framework. Our approach bridges the gap between evolving contemporary concepts on equity and the structural design of T32 programs, ensuring that our training program not only aligns with these values but also sets a precedent for future adaptations of similar initiatives. Sharing lessons learned (Kross et al., 2020) with the broader community can foster a collective effort to further strengthen the impact and inclusivity of these programs in addressing HIV workforce disparities.

This commentary paper aims to delineate the strengths and weaknesses of CHANGE, a T32 program at a university in South Florida. Drawing from the perspectives of both its inaugural cohort of trainees and the program’s directors who oversee and deliver the program, the paper offers insights and recommendations for advancing the effectiveness and impact of such training initiatives. Experiences and insights of both trainees and leadership were meaningfully gathered iteratively through a combination of structured discussions, collaborative writing sessions and reviews of drafted content during our weekly meetings, ensuring time was set aside to review and refine drafts. The trainees each focused on and drafted specific sections, the first author synthesized the sections to harmonize the flow, and the directors provided iterative edits and feedback.

### Overview of CHANGE

The directors (e.g. experienced faculty members and researchers with a history of involvement in NIH-funded research and mentoring and training doctoral students, post-doctoral fellows, and junior faculty) were vividly aware of gaps through ongoing engagement with community partners and leadership in HIV community engaged efforts, mentorship of minoritized scholars, and personal journeys to conducting impactful health equity research. Hence, they applied to the NIH-T32 mechanism after identifying a critical need across our institution and the Center for HIV and Research in Mental Health (CHARM) network to develop the next generation of HIV researchers equipped to tackle emerging challenges in the field. A longstanding HIV T32 in psychology had been in place for decades (1994–2013); however, applying for a new T32 required addressing today’s critical issues, especially the intersecting impacts of HIV and mental health on Black, Latine/x, and LGBTQ+ communities facing deep inequities (NIH RePORTER, 2013). This new approach required training scholars dedicated to serving these communities through community-engaged research and providing trainees with the content knowledge and methodological skills needed for impactful work. The T32 framework leveraging strengths and approaches (Patel et al., 2023) from the Departments of Public Health Sciences and Psychology provided an ideal structure for fostering interdisciplinary training, mentorship, and skill development, aligning with our goal to invest in future leaders in HIV research. This program allowed us to strategically address gaps in expertise within the university and community.

CHANGE is an interdisciplinary, innovative, and timely training program which focuses on both mental health and public health. The program is specifically designed for predoctoral and postdoctoral scholars (particularly those in the field of HIV behavioral science) committed to addressing HIV and mental health inequities in Black, Latine/x, and LGBTQIA+ communities, with inclusion criteria aligned with NIH T32 guidelines. Eligible candidates included individuals committed to improving the lives of marginalized groups disproportionately impacted by HIV and mental health, as well as those with content interest and potential experience, through research or community involvement in HIV and mental health and in related fields. Additional details on the eligibility requirements can be found in the NIH-T32 Funding Opportunity Announcement (Department of Health and Human Services, 2020). The training program fully funds and supports the professional development of trainees.

CHANGE training goes beyond conventional scientific training by investing in the next generation of scientists aiming to develop careers dedicated to addressing HIV and mental health inequities. The program provides rigorous training in research design/methods, CBPR, and implementation science. CHANGE also emphasizes the importance of inclusive mentorship and building professional networks. Inclusive mentorship, within the context of our program, is characterized by fostering a supportive and diverse environment where mentorship extends beyond traditional boundaries (Brown & Montoya, 2020; Filingeri et al., 2023) to ensure the holistic development of each trainee. This mentoring approach reflects mentors’ awareness of both their own experiences with institutional/systemic challenges and the impact of those challenges on the scholars they mentor, embodying a dual consciousness regarding institutional impact (Gandhi & Johnson, 2016; Gwayi-Chore et al., 2021; Jenkins, 2022). The dual consciousness that mentors possess comes with the understanding of the ‘hidden curriculum’ (i.e. the unspoken expectations and norms within academic environments) (Park et al., 2023; Webb, Arthur, et al., 2022a), the pressures of being the ‘first and only’ in predominantly white or heteronormative spaces (Jones et al., 2022), and the daily and cumulative tolls of intersectional microaggressions and invalidations (Archuleta et al., 2024; Fu et al., 2024). Engaging with these concepts allows mentors to understand and validate trainees’ lived experiences, fostering trust and helping trainees navigate structural challenges while advancing their academic and professional development. In this environment, mentors create a safe space within seminars and facilitate open discussions on challenging topics that might not be as freely addressed or discussed in other settings. All training is grounded in a culture of vulnerability, encouragement, and resilience.

**Figure 1.**
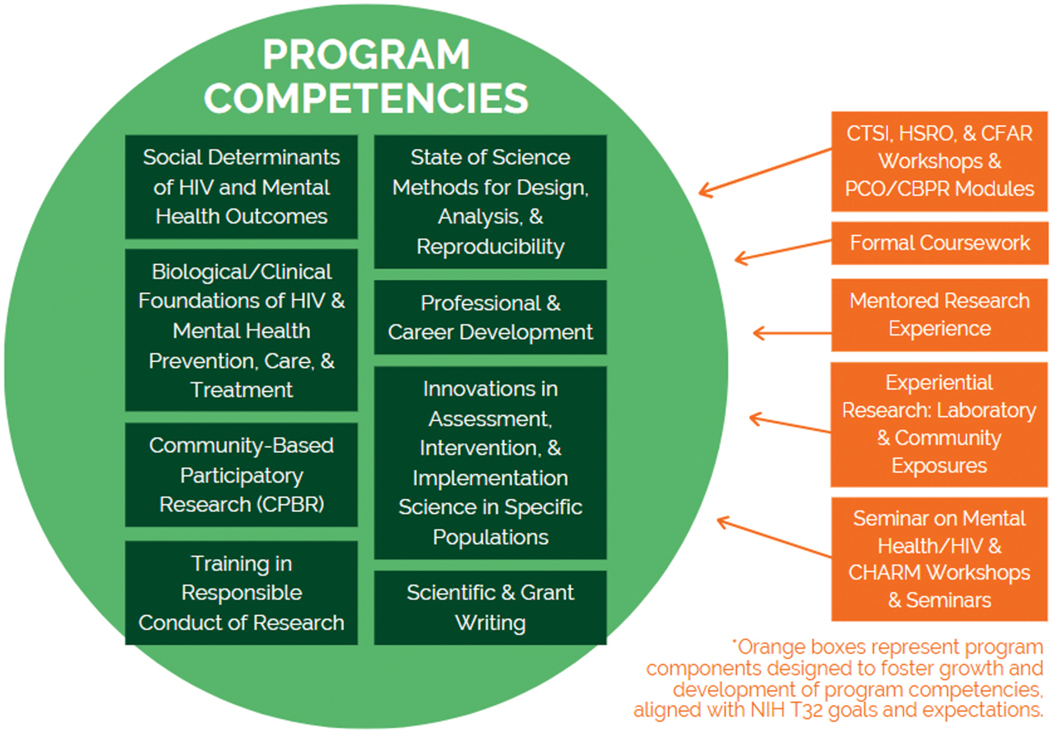
CHANGE program competencies. *CTSI* = Clinical and Translational Science Institute; *HRSO* = Human Subjects Research Office; *CFAR* = Center for AIDS Research; CHARM = Center for HIV/AIDS Research and Mental Health.

Eight core competencies are covered in the training program (Figure 1). Program competencies are honed through a comprehensive framework that includes coursework, workshops, experiential training integrated within both research labs and community settings, complemented by systematic mentorship. Key centers, such as CHARM, Miami Clinical and Translational Science Institute (CTSI), Human Subject Research Office (HSRO), and the Miami Center for AIDS Research (CFAR), serve as invaluable resources for CHANGE. Leveraging their established infrastructure and expertise, CHANGE aims to enhance connectivity among researchers and foster breadth and depth within the focused training program. By tapping into these centers’ resources, a robust framework for HIV research was established, particularly in the context of mental health by capitalizing on existing infrastructure to support CHANGE effectively.

**Figure 2.**
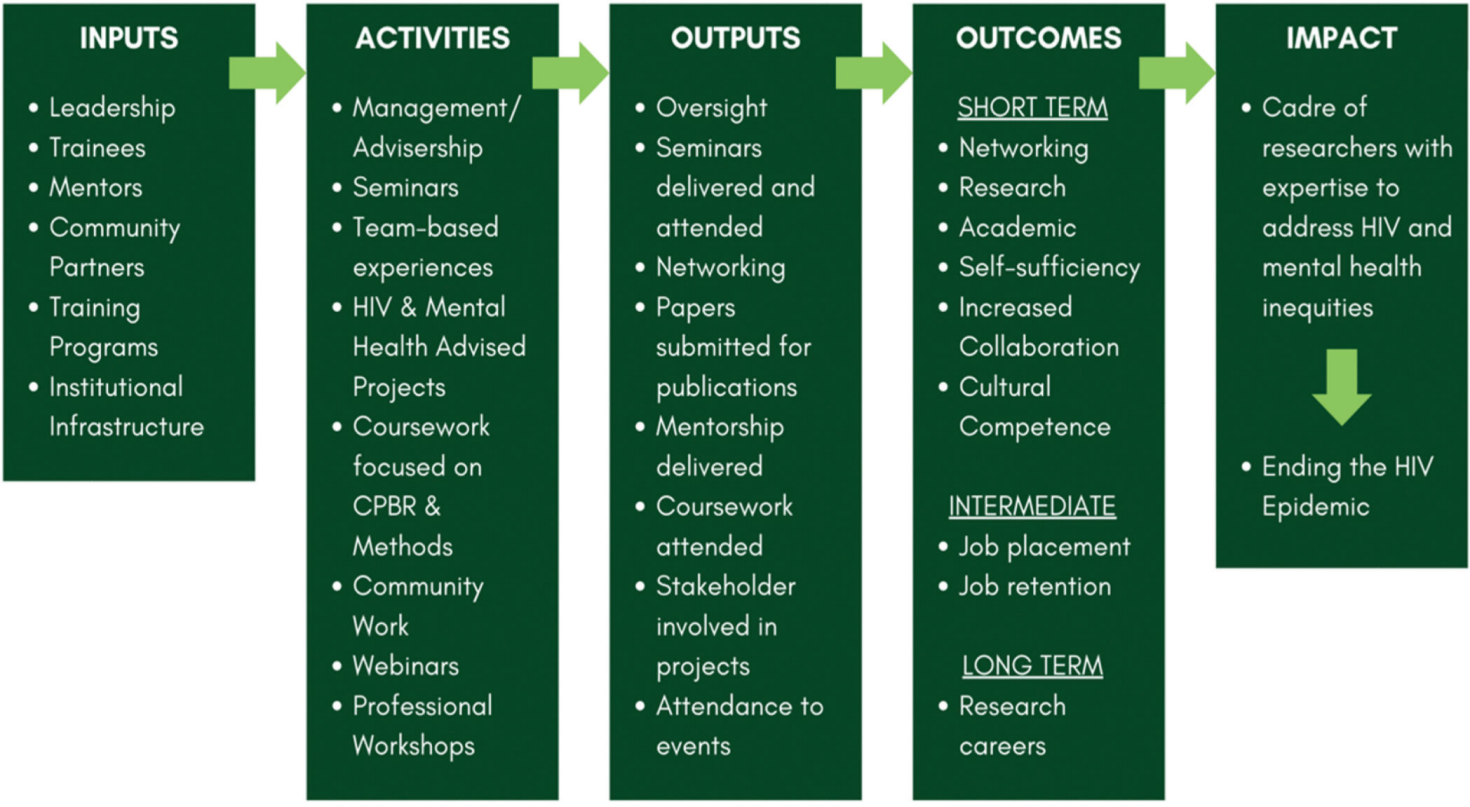
CHANGE logic model.

Essential training areas include social determinants of health, community-based participatory research, research and intervention design, and in-depth analysis of the intersection between HIV and mental health. Trainees engage in a community project, where they cultivate partnerships with community organizations serving Black, Latine/x, and LGBTQIA+ populations in South Florida. The program’s holistic approach (Figure 2) integrates various elements, including university leadership, trainees, mentors, community partnerships, and institutional infrastructure. Through a range of activities such as seminars, team-based experiences, and community engagement, the program aims to foster networking, research skills, academic growth, self-sufficiency, and cultural competence among participants. Ultimately, the program seeks to cultivate a cadre of researchers equipped to address HIV and mental health disparities, contributing to efforts to end the HIV epidemic.

Scholars gain insights into HIV and mental health through frameworks such as intersectionality and trauma-informed pedagogy. Workshops focus on understanding the unique impact of HIV on various minoritized populations and the compounded effects of interlocking systems of oppression on individuals. Trauma-informed pedagogy is actively applied through community partnerships to ensure that educational approaches are sensitive to the unique challenges and lived experiences of individuals trainees serve. Key components from CHANGE such as structured seminars, interdisciplinary mentorship, and community partnerships can be adapted by other institutions to meet their specific needs. Table 1 highlights these program components and details their implementation as done for CHANGE. We tailored these elements by leveraging existing strengths within the CHARM network and across public health, psychology, and related departments, fostering cross-disciplinary research. This approach offers a flexible framework that other institutions can modify to align with their unique contexts and objectives.

#### Strengths

CHANGE features several notable strengths. Firstly, CHANGE leverages Miami’s unique context, providing scholars with opportunities to engage with the city’s rich cultural landscape and diverse communities in their research and training endeavors. Secondly, the program benefits from the scholarly rigor, lived expertise, and diversity of its trainees, fostering an inclusive learning environment that encourages cross-cultural exchange and collaboration. Thirdly, the program’s curriculum and key features are carefully designed to address the specific needs and challenges of scholars, offering a comprehensive and tailored training experience. Finally, the program’s leadership and mentorship networks provide invaluable guidance and support, nurturing the professional growth and development of scholars throughout their journey.

### Miami’s rich cultural context

Miami-Dade County (MDC) represents one of EHE jurisdictions with high HIV inequities found among Black and Latine/x individuals (Zang et al., 2023). In MDC, the population is predominantly Hispanic or Latino (69.1%), followed by White individuals not of Hispanic or Latino origin (13.8%), and Black or African American residents (17.1%) (U.S. Census Bureau, 2022). A substantial portion of the population is foreign-born, accounting for approximately 54.0% of residents (U.S. Census Bureau, 2022). According to UCLA’s Williams Institute, the Miami metropolitan area has approximately 214,000 LGBTQIA+ residents, representing 4.5% of the population (Conron et al., 2021). CHANGE is therefore positioned within reach of the very communities most impacted by the HIV epidemic and whose voices are underrepresented in research. The program leverages the unique cultural diversity present in MDC, through collaborations with local organizations serving diverse populations (e.g. Latine/x MSM, recently arrived immigrants, Haitian communities) to support trainees as they develop strategies that are locally relevant yet have the potential for impact across other EHE jurisdictions.

### The cohort of trainees

CHANGE include pre- and post-doctoral trainees in public health and psychology disciplines, reflecting the diverse expertise and perspectives needed to address complex health disparities. The intentional application and selection process aimed to recruit scholars with a commitment to addressing HIV and mental health inequities impacting Black, Latine/x, and LGBTQIA communities, thereby advancing the HIV research agenda. While not a specific selection criterion, CHANGE inherently attracted individuals who were representative of these communities themselves and have lived experience as well as a profound connection to affected communities. Previous studies have highlighted the transformative impact of diversifying research initiatives (Irie et al., 2023; Stoff et al., 2023). Recognizing that traditional human resource (HR) processes – such as rigid eligibility filters and automated screenings – can limit prospective trainee access to these opportunities, CHANGE supplemented formal recruitment with webinars, direct communication, and consultations/meetings with co-directors outside HR oversight. Our advisory board also played a key role by providing insights on applicants, guiding selection, and ensuring the process remained equitable. While managing these parallel efforts was complex on our end, they ultimately made the process smoother and transparent for applicants.

Enhancing the diversity of the HIV research workforce brings advantages such as innovation in research and development, increased societal impact, and the expansion of research agendas to include topics relevant to people living with HIV (Irie et al., 2023). For example, trainee projects focused on adapting and developing a status-neutral structural evidence-based ‘employment as HIV prevention’ intervention for newly arrived Spanish-speaking Latinx/e immigrants, understanding the impact of intersectional adversities on cardiovascular disease risk among Black women living with HIV, examining facilitators and barriers to COVID-19 vaccine uptake among Black women living with HIV, utilizing machine learning to understand the influence of racism on HIV outcomes, and supporting HIV care continuity for highly mobile and displaced communities, among others (Boga & Dale, 2022; Casanova et al., 2023; Casanova et al., 2024; Forney et al., 2024; Jaramillo & Harkness, 2023; Jaramillo et al., 2024; Londono et al., 2024; NIH RePORTER, 2024a, 2024b; Schmidt et al., 2024; Shahid & Dale, 2024; Wright et al., 2022). Ultimately, CHANGE aims to foster diverse perspectives and research agendas through its diverse cohort, ultimately driving innovation and addressing critical health issues related to HIV.

### Curriculum and key features

CHANGE embeds and facilitates trauma-informed pedagogy (Brown et al., 2021) and intersectionality theory (Collins et al., 2021; Crenshaw, 1989) through several core curricular components designed to create a supportive, inclusive learning environment for trainees. To support readers interested in incorporating trauma-informed pedagogy and equity-focused mentorship into their programs, Table 2 provides key elements of our curriculum. As shown in Table 2, these components – ranging from trauma-informed facilitation and co-created seminar content to structured debriefing, community-engaged research, and routine feedback mechanisms – enable us to build empathy, resilience, and cultural responsiveness within the training process. The training program embodies principles of mutuality, acknowledgement of existing trauma (individual, lived, and structural), transparency, avoidance of re-traumatization, trustworthiness, peer support, and collaboration, which are essential for carrying out community-based work (Brown et al., 2021). Each element supports trainees in developing the skills and sensitivities necessary for equity-focused research, building a foundation that not only prioritizes mental health and community-centered values but also prepares trainees to implement trauma-informed approaches into their current and future professional environments. The initial meeting exemplified this ethos, breaking barriers through powerful personal narratives, fostering vulnerability, support, and understanding from the outset.

Another notable aspect of CHANGE is our approach to weekly seminars, aligning with the principles of transparency of teaching and learning (Winkelmes, 2023). We collaboratively design seminar presentations tailored to meet the current professional development needs and advancement goals of trainees, while simultaneously ensuring delivery of core content. Careful consideration is given to the selection of presenters to ensure that diverse perspectives are represented. Trainees have increased input over the content and speaker selection, fostering learning experiences that reflect the diverse backgrounds, research interests, and career stages within the fields of HIV prevention, treatment, and mental health research.

The CHANGE program conducts weekly seminars remotely, while other key components, such as mentorship, research in labs, and coursework, are conducted through in-person interactions. This hybrid approach offers a significant advantage, particularly considering the diverse mix of pre-docs, post-docs, and the busy schedules of directors. The remote setup facilitated social connectivity and innovation, fostering a safe space where diverse emotions and conversations unfolded. Deliberate approaches such as informal conversations, debriefing sessions, one-on-one mentoring, and group writing contributed to the success of the CHANGE training across a connected virtual space.

### CHANGE leadership and mentorship networks

The T32 is led by three directors who bring complementary expertise in (a) health equity and community engaged research, intersectionality, and mentoring minoritized scholars, (b) public health education, psychiatric and substance use clinical trials, and working with Latinx populations, and (c) HIV behavioral science methodology, biostatistics, study design, implementation science, and machine learning. In addition to three directors – representing diverse disciplines, professional identities, and cultural backgrounds – who provide weekly mentorship to trainees, CHANGE ensures access to diverse affiliate faculty mentors who specialize in various fields such as medicine, public health, psychology, sociology, biostatistics, and epidemiology. Change leadership and mentorship networks are connected to CHARM, which stands out as a unique hub for HIV and mental health research and training in the state of Florida, particularly around behavioral research. The comprehensive mentoring structure acknowledges the need for multiple mentors to address diverse expertise, various perspectives, knowledge gaps, and power dynamics. The program also acknowledges and aims to alleviate the disproportionate burden of mentorship that is often shouldered by senior women of color, a phenomenon described in recent scholarship (N. E. Brown & Montoya, 2020), by implementing strategies for equitable distribution of mentorship responsibilities.

To promote equitable distribution of mentorship responsibilities, the CHANGE program implements a collaborative mentorship model. For example, during the fall semester, trainees navigated the program’s tiered mentorship structure by rotating through individualized meetings with each of the three program directors on a regular basis. In one session, a trainee focused on grant writing strategies and received feedback on a draft NIH K01 application. In addition to ongoing mentorship from CHANGE Directors and their primary mentors on the application, this trainee also received expert consultation on recruitment approaches and study design and accessed CHARM’s Grant Peer Review service, ultimately submitting a successful award. In another session, a different trainee refined an outreach strategy for a community project in collaboration with a local HIV service organization. Alongside these rotations, the same trainee connected with their affiliated mentor from the public health department, selected for their expertise in mixed-methods research, as well as a mentor from the community organization where they volunteered, who offered culturally grounded perspectives on the project’s potential impact. To better ensure that no mentor was disproportionately burdened, the program directors met regularly to coordinate their mentoring efforts. Primary and affiliate mentors also participated in annual private meetings with the directors to discuss trainee progress and align mentoring strategies as needed. Throughout the process, trainees and their mentors completed periodic surveys to monitor the mentoring relationship and track professional development goals. This model not only helps to distribute mentorship responsibilities but also provided trainees with a diverse, interdisciplinary support network that enriched their learning experience. However, it is still important to acknowledge that efforts may not completely resolve all the distribution challenges as mentees may call on, seek out, and solicit mentorship and guidance from mentors whose research, lived expertise, and positionality are more proximal to their own. The CHANGE mentorship approach actively works to aligns with current models, such as the Justice, Equity, Diversity, and Inclusion Academic Mentors (JAM) Council, which provide culturally responsive and anti-racist strategies through structured mentorship to support the retention, promotion, and career satisfaction of underrepresented faculty in academic medicine (Bath et al., 2022).

#### Challenges

CHANGE encountered multifaceted challenges ranging from individual, program, and systemic levels, impacting both scholars and mentors. While some of these challenges directly impacted CHANGE, other challenges reported aligned with those previously documented by other training programs, highlighting the relevance of systemic issues within academia and the broader structural racism embedded in communities that significantly impact the learning trajectories of minoritized individuals. Nonetheless, we deemed it valuable to incorporate them, distinguishing between those applicable to our program and those drawn from the broader literature on similar training programs. They comprise the underrepresentation of minoritized scholars, limited program investment, systematic inequities, issues related to mentee selection and mentorship, and limited trauma-informed support for scholars.

### Underrepresentation of minoritized scholars

The underrepresentation of minoritized scholars across health research programs poses a challenge for achieving a diverse HIV and mental health workforce (Nowotny et al., 2020; Singh et al., 2018). While CHANGE made notable strides in attracting, recruiting, and retaining a diverse cohort of scholars, underrepresentation remains a pervasive challenge across similar programs (Filingeri et al., 2023; Stoff et al., 2023; Torres Acosta et al., 2023). Scholars in the initial cohort faced various challenges before joining CHANGE, such as limited access to educational resources, microaggressions within academia, and discrimination. While these experiences may have enhanced their resilience, they also weighed heavily on them once in the program. The rigorous demands of CHANGE have both bolstered some scholars’ trajectories and unveiled existing gaps in their developmental trajectories, highlighting different needs for each. Balancing internships, academic/professional commitments, and personal responsibilities can overwhelm scholars, underscoring the crucial need for additional support to effectively manage stress and expectations throughout their journey.

Although many training programs aim to cultivate a diverse and inclusive research community, a significant gap exists in achieving representation of scholars with lived experiences who are often a part of communities who have been socially marginalized (e.g. Black, Latine/x, LGBTQIA+ individuals from a lower socioeconomic status) (Esparza et al., 2022). This challenge stems from various factors, including historical disparities in educational opportunities, recruitment biases, and a lack of targeted support structures (Widge et al., 2023). For CHANGE, overcoming these challenges demanded a fundamental shift in the culture of inclusivity and diversity, which is recommended by researchers (Singh et al., 2018). Underrepresentation not only hinders the individual opportunities of minoritized scholars but also limits the potential for broadening the scope of research and addressing health disparities effectively for different populations impacted by HIV (Nowotny et al., 2020; Singh et al., 2018).

### Limited investment by funders in equity and participatory approaches

The current system of research funding for trainees highlights troubling disparities which were also experienced by our CHANGE scholars, particularly around issues of equity and diversity (Garrison & Deschamps, 2014; Pohlhaus et al., 2011; Steinbach et al., 2018). For example, in a study on sex differences in NIH award programs, researchers found that while women and men were generally equally successful at all career stages in securing NIH funding, longitudinal analysis revealed that men with previous experience as NIH grantees had higher application and funding rates than women at similar career points (Pohlhaus et al., 2011). Despite women receiving larger R01 awards on average, men consistently held more R01 awards throughout their careers, highlighting the need for further action to address these disparities (Pohlhaus et al., 2011). Similarly, in a more recent study on NIH predoctoral fellowships, researchers found that between 2001 and 2020, the growth rate of diversity F31 fellowships (i.e. fellowships that support underrepresented predoctoral and post-doctoral-level trainees in biomedical science) was 89% lower than that of general F31 fellowships, and the number of new F31 diversity awards remained stagnant since 2010 despite a diversifying graduate student population (Nguyen et al., 2022). Researchers attributed the gap to slower growth in diversity F31 applicants, decreased award rates, or underrepresented applicants opting for general F31 fellowships or alternative funding sources. Their findings highlighted persistent challenges experienced by our scholars, emphasizing the necessity for additional support, mentorship, and initiatives beyond the program’s efforts to facilitate equitable research funding and career advancement (Nguyen et al., 2022).

A challenging funding climate, marked by inconsistent financial support and a lack of a clear pipeline for opportunities, poses a significant barrier for minoritized scholars (Garrison & Deschamps, 2014; Meyers et al., 2016; Nguyen et al., 2022). T32 programs like CHANGE, which play a vital role in scholar development and fully fund scholars (e.g. monthly stipend, tuition, and conference travel), still often grapple with limitations in additional funding and resources, largely stemming from the broader funding climate and resource allocation practices within academic and research sectors (Steinbach et al., 2018). This constrained financial landscape directly impacted our program capability and the number of trainees that could be supported, thereby limiting the program’s ability to nurture a broad spectrum of talents. Recent studies underscore these challenges, revealing a disproportionate distribution of funding, with only a small number of researchers receiving a substantial share, while underrepresented scholars and those engaged in community-based participatory and equity work encounter significant hurdles in securing research funding (Nowotny et al., 2020; Zambrana et al., 2015). This unequal allocation of resources not only hinders the progress of vital research in these areas but also perpetuates systemic disparities within the academic and scientific communities, making it imperative to address these inequities to achieve a more inclusive and equitable research landscape. Although underrepresented students, staff, and faculty are often driven by an inherent motivation to innovate in areas aligned with their lived experiences, their research interests – though deeply relevant – are frequently undervalued within academia (Settles et al., 2021). This leaves them disproportionately burdened by the dual expectations of both colleagues and their communities to lead these efforts, while also facing challenges in securing the funding and institutional support needed to pursue meaningful change, reflecting a persistent tension between the drive for innovation and the inertia of resistant institutions. Addressing these challenges requires intentional institutional equity strategies that support and sustain these scholars’ efforts to advance transformative, HIV equity-focused research.

### Systemic inequities

Training programs often prioritize equipping scholars with knowledge, tools, and skills to conduct research but frequently overlook the inclusion of spaces for sharing experiences, addressing trauma, and weaving personal histories into the training process that could be particularly beneficial for minoritized scholars (Esparza et al., 2022; Wright et al., 2023; Zhou & Louie, 2022). Failing to incorporate these aspects into T32-training programs could pose a challenge in fostering truly inclusive and supportive training environments. Systemic issues perpetuating inequities in academia and grant funding for minoritized scholars demand heightened focus to prepare scholars for the challenges they will face in the real world (e.g. limited early access to advanced training and opportunities, discrimination and microaggressions, feelings of isolation due to the absence of peers and senior mentors, restricted access to early funding, community and personal financial pressures) (Singh et al., 2018; Widge et al., 2023). Some of these systematic inequities were experienced by the scholars in our first cohort, extending beyond CHANGE including tokenization, limited grant-writing support, challenges accessing tailored funding opportunities, and insufficient guidance in navigating grant applications. Such biases manifest in biased selection processes, unequal access to resources, and the absence of inclusive institutional policies, emphasizing the need for comprehensive reform to level the playing field for scholars from all backgrounds (Peña et al., 2023).

### Mentor selection and mentorship

The process of providing effective mentorship (via departments of origin and extended networks) to mentees within CHANGE mirrored issues identified in the literature, including the lack of consideration for cultural factors in mentoring processes, limited training in culturally sensitive mentorship, and the overreliance of matching based primarily on expertise, which can hinder the success of minoritized scholars (Brunsma et al., 2017; Pfund et al., 2022; Widge et al., 2023). The potential harm to minoritized scholars from unrecognized lived experiences and trauma due to intersectional oppression can exacerbate existing barriers to their academic and professional success (Zhou & Louie, 2022).

Our challenges encompassed more than just matching scholars with mentors; they involved addressing the disparities often present within mentorship relationships (Brunsma et al., 2017; Martinez-Cola, 2020). For instance, persistent gaps in mentoring programs are evident for minoritized scholars in research careers stemming from relationships with mentors who may unknowingly hold biases or struggle to accommodate their trainees’ diverse needs (Widge et al., 2023). Such biases affect how mentors effectively engage with and guide trainees, as well as the overall success of the program in fulfilling its objectives (Widge et al., 2023).

These challenges underscore the necessity for mentorship programs to transcend mere expertise matching, demanding mentors who can engage in deep reflection and personal growth. To overcome difficulties in encouraging mentor participation and maintaining their interest in the mentorship process (Singh et al., 2018), effective mentorship training should prioritize enhancing qualities like humility and fostering mutual learning and development among mentors. Further efforts are needed to ensure mentorship programs sufficiently address the diverse needs of mentees and cultivate meaningful mentor-mentee relationships. Addressing these areas within CHANGE and beyond highlights the current need for best practices in mentoring minoritized researchers.

### Limited trauma-informed support

Scholars within CHANGE encountered diverse forms of trauma, particularly because they belonged to underrepresented groups within academia and broader society. Their lived experiences mirrored the wider societal issues and systemic inequities that other minoritized scholars confront daily and illustrated how these challenges manifested themselves as unique stressors within academia (Nowotny et al., 2020; Ramasubramanian et al., 2021). For instance, minoritized scholars frequently confront a myriad of obstacles rooted in structural biases present within research training and funding institutions, leading to disparities in both representation and the availability of opportunities (Widge et al., 2023). Many minoritized researchers have also had to contend with various forms of trauma, whether originating from early-life adversity or the enduring experience of microaggressions at various points along their academic and professional journeys (Widge et al., 2023).

CHANGE aims to address trauma-related challenges by incorporating trauma-informed pedagogy (Brown et al., 2021; Ramasubramanian et al., 2021) to support the well-being and success of scholars. While elements of trauma-informed pedagogy were integrated into the seminars such as facilitating discussions on resilience and coping strategies for dealing with trauma-related challenges, the complexity of trauma experienced by scholars necessitates further support. We realized that it is insufficient for the program’s directors alone to offer a safe space if the institution lacks mechanisms to address and process traumatic events, such as microaggressions effectively. Addressing these challenges requires support across multiple levels, including the course, lab, seminar, graduate program, school, and institutional levels. Additionally, integrating these efforts into larger institutional infrastructure, with a focus on trainees and expanding access to safe spaces, are currently needed to address the unmet needs of scholars and support their mental health and coping skills.

#### A call to action to drive CHANGE

To effectively support the development of pre/post-doctoral HIV training programs aimed at nurturing the research and professional trajectories among those dedicated to serving underserved communities, we need to implement additional strategies to ensure long-term sustainability. Both public health and psychology are interdisciplinary fields that aim to improve the health and wellbeing of all individuals and communities impacted by HIV, and we cannot achieve this goal without the contributions of a diverse HIV and mental health workforce.

While the primary aim of the CHANGE program focuses on addressing disparities within academic HIV science, the program also provides significant benefits to trainees who may later diverge to alternative career pathways including roles in clinical practice, non-academic research settings, local government, and community-based organizations. Through training in applied research methods, public health advocacy and leadership, and multi-sector partnerships, trainees gain practical skills applicable to non-academic environments. The program’s focus on community engagement and culturally grounded research equips trainees to work effectively with diverse populations impacted by the HIV epidemic, preparing them for leadership roles across both academic and non-academic settings. This call aims to optimize the impact of training programs on developing an HIV workforce equipped to address structural inequities and shaping the future of academic, clinical, and community efforts.

We present a framework and recommendations from CHANGE to enhance the reach, effectiveness, and sustainability of HIV-focused training programs across policy, institutional, and community levels. We hope that institutions involved in establishing HIV-focused T32 programs heed the call to action by developing strategic plans led by designated taskforces to ensure concrete steps for practical implementation align with the outlined objectives. By prioritizing these initiatives, we can create a more equitable and empowering training environment that uplifts scholars and equips them to make meaningful contributions within their communities.

### Call to action 1: develop innovative and tailored pedagogical techniques for future trainees

To foster an inclusive learning environment, academic institutions should implement innovative and tailored pedagogical techniques, such as trauma-informed curricula (Cavener & Lonbay, 2024) delivered bidirectionally to support trainees’ diverse needs. It is crucial for faculty and staff to be equipped with tools to champion diversity and acknowledge the impact individual and institutional-level discrimination has on the well-being of underrepresented scholars and communities they aim to serve. This approach requires addressing implicit biases, fostering culturally responsive pedagogy and mentorship, and cultivating a welcoming and supportive atmosphere for all trainees, ensuring their well-being and success (Gandhi & Johnson, 2016).

Considering the implementation of these initiatives raises the question of whether faculty should demonstrate proficiency in these approaches before engaging in them. This underscores the importance of more than just good intentions. Without the requisite skills or training, initiatives like institutional culturalized mentorship training (Pfund et al., 2022) could support faculty in fostering culturally responsive pedagogy and mentorship. Therefore, university leadership should prioritize these efforts, as faculty may not give due attention to them without institutional backing.

Programs should create a safe and inclusive space for trainees and mentors to process HIV-related information, acknowledging that some scholars may have personal experiences with HIV that make the material emotionally challenging. Others may experience vicarious trauma due to their personal or professional connections to HIV. This environment could allow for open discussion, reflection, and the addressing of any concerns or issues that arise, ensuring that all participants feel supported and heard. The directors of programs could ensure that mentorship initiatives (Nearing et al., 2020) are inclusive and intentional, foster meaningful connections, and leverage the strengths of mentors. They could consider organizing mentor mixers to facilitate networking, address mentor commitment to the program’s goals, and convey the program’s expectations regarding addressing the structural barriers that impact the mentor/mentee relationship in academia. Mentorship programs could be designed with sensitivity to the lived experiences of minoritized scholars and include strategies to address potential intersectional oppression-induced trauma due to participation in the program and broader academia.

### Call to action 2: develop strategies for the recruitment of underrepresented scholars

A culture of inclusion begins with proactive recruitment (Dawkins, 2021; Greenberg et al., 2023; Magnus et al., 2023) and unwavering support for individuals from underrepresented backgrounds. This involves adopting a more holistic approach to evaluating applicants, valuing non-traditional paths, and recognizing lived experience as expertise, thereby moving beyond traditional metrics in the selection process. Lived experience underwrites impactful original research directions in several crucial ways, such as generating hard-hitting and credible research questions or hypotheses grounded in real-world challenges (Beames et al., 2021; Mehrabadi et al., 2024). Researchers with lived experience are better equipped to identify and analyze structural and systemic forces that are often understudied (Bath et al., 2022; Mehrabadi et al., 2024; Muhammad et al., 2015). In qualitative research, lived experience strengthens rigor and trustworthiness by fostering deeper connections with participants and enabling more nuanced data interpretation (Morrow, 2005). These insights drive research that is both rigorous and socially responsive, aligning scholarly inquiry with the lived realities of communities most affected by the HIV epidemic. Selection committees should therefore be trained to recognize and prioritize lived experience as an essential asset in prospective candidates, appreciating their potential to generate high-impact research grounded in community connection and lived expertise.

Outreach and recruitment in traditional avenues are not enough; directors and institutions must be intentional and creative in their efforts to recruit underrepresented scholars. Recruitment should extend through community organizations, professional networks, interest groups for minoritized scholars, and academic institutions for historically Black and Latine/x minority serving institutions. Employing various outreach methods such as social media outlets, events, email, informational sessions, and word of mouth, and implementing referral incentives (e.g. encouraging current participants, alumni, or faculty members to refer qualified candidates by offering referral incentives or rewards) could enhance the visibility of opportunities. Additionally, ensuring a clear and streamlined application process facilitates accessibility and encourages diverse participation.

### Call to action 3: evaluate and invest in training programs

Given the persistent challenges posed by HIV which remains a pressing national health concern particularly in the southern states, and the declining HIV provider workforce (Armstrong, 2021) in the US, prioritizing investment in training programs becomes increasingly vital for recruiting, retaining, and supporting a diverse and well-equipped workforce capable of addressing HIV priorities. To reach the goal of ending the HIV epidemic by 2030, it is essential to expand the availability of T32 programs and ensure their effectiveness to sustain a robust pipeline for the HIV workforce. The NIH and universities housing training programs should conduct annual evaluations of T32 program goals, trainee achievements and trajectories as a metric of success and failure (Jenkins et al., 2020; Kross et al., 2020). These evaluations should inform funding decisions for federal, academic, and other research institutions in contributing to a stronger and more diverse HIV workforce. Analyzing the training landscape at both local and national levels and engaging program officers to inform early career trainees, particularly minoritized scholars, about training and career development opportunities, including those that promote diversity and offer tailored training approaches specific to their needs could be useful. In 2023, 329 T32 training grants were awarded according to NIH (National Institutes of Health, 2024). These programs are not exclusive to HIV and mental health and systematic evaluations of these programs are limited (Kross et al., 2020). Mechanisms to evaluate an institution’s commitment to trainee success are needed (Koethe et al., 2023; Pitpitan et al., 2023), including coordination with the training resources and mechanisms of the NIH geared towards complementary training opportunities.

Increased coordinated institutional financial commitment from both the NIH and academic institutions (Jenkins et al., 2020) will be essential to bridge funding for a career pipeline, facilitating the successful transition of trainees into research independence, industry, or other HIV workforce paths. This support will ensure that host universities, program directors, mentors, and scholars have the necessary resources to facilitate this transition effectively. For example, a designated fund could be allocated for incentives or rewards, to acknowledge the often-unpaid service provided by directors and mentors, especially those from underrepresented backgrounds or those working with trainees from similar backgrounds who are committed to fostering diversity. Additionally, a negotiated agreement between funding institutions and universities could be established to provide financial support during the transitional period for trainees nearing the end of their training thereby preventing any gaps in trainee career progression.

### Call to action 4: amplify institutional resources for professional development

We call upon institutional leaders and stakeholders across academic bodies, research institutions and grant-funding organizations to endorse the mission of enhancing institutional resources for the professional development of minoritized scholars (Fernandez et al., 2023; Hemming et al., 2019; Jenkins et al., 2020; Pitpitan et al., 2023). Institutions must bolster their support for T32 training grants by providing the essential resources and infrastructure for effective research training. This encompasses access to state-of-the-art laboratory and clinical facilities, proficient data management tools, and avenues for professional development (Hemming et al., 2019). Additionally, universities lacking these resources should establish connections with other institutions to ensure that minoritized scholars have the necessary support and opportunities for career advancement. The goal is to create an environment where trainees are supported and empowered to thrive.

Ensuring the sustainment of the trainee pipeline requires sustained mentorship and career development support, fully leveraging institutional resources. Beyond recruitment efforts, it’s essential to establish infrastructure that fosters success, and offers resources for tangible research and field opportunities, training in grant and scientific writing, collaboration skills, and network opportunities. These may include participation in symposiums, conferences, and webinars where trainees can showcase their work, connect with other professionals in the HIV workforce, and cultivate new mentorship or collaborative opportunities for research, projects, and future career opportunities.

Mechanisms of support could encompass dedicated funding to pay mentors, ample professional development opportunities, access to essential resources such as funding and research facilities, along with robust support and resources to support the mental health and work-life balance of predoctoral and post-doctoral scholars.

### Call to action 5: enhance resources and funding for community health equity research

Building partnerships with community organizations and stakeholders (Akintobi et al., 2023; Ibáñez-Carrasco et al., 2020; Jenkins et al., 2020; Sutton et al., 2021; Wallerstein et al., 2020) actively involved in HIV prevention, care, research, and treatment at the grassroots level is crucial for sustaining the impact and relevance of HIV-focused T32 programs, ensuring that they remain responsive to the needs of the communities they serve. This will also require advocacy at the university level to focus attention towards funding support for research topics that delve into health disparities research and the determinants of health that are prevalent, understudied, and those that challenge the current status quo to drive innovation and social change for the betterment of underserved communities impacted by HIV (Greenwood et al., 2022; Marshall & Cahill, 2022; Veale et al., 2022). Institutions should prioritize research aimed at dissecting the root causes of health inequities and identifying effective community-informed interventions to mitigate disparities.

Establishing funding programs with a focus on collaborations between trainees and HIV community-based organizations (CBOs) and clinics are needed. It is important that trainees are exposed to community-based HIV work in communities with HIV disparities and have opportunities to understand how their work could be in partnership with the goals of community organizations and clinics. The conditions must facilitate true CBPR, enabling community partners and trainees to engage in mutually beneficial and equitable partnerships. This entails community organizations co-leading grant opportunities, research design, budgets, and dissemination efforts, ensuring that the research reflects and addresses the real needs and priorities of the community.

We need concrete steps to ensure that pre-doctoral and post-doctoral training programs in public health and psychology become more equitable, diverse, and supportive. This shift will not only empower individual scholars but also strengthen our healthcare system and better serve our communities. By taking coordinated and deliberate actions, we can pave the way for a more inclusive and equitable future for scholars and researchers alike.

## Conclusion

This commentary offers an examination of the strengths, challenges, and recommendations pertinent to an NIH-T32 training program to drive CHANGE forward for mental health and HIV research. The program embodies a holistic approach for cultivating a supportive and inclusive environment to promote the academic and professional success of trainees. However, additional opportunities to further realize the vision of this training program still remain, including furthering institutional investment in mentorship processes and funding agencies providing for consistent and increasing grant opportunities for underrepresented scholars. Moving forward and to better support current and future T32 programs, widespread support across various institutions and fund organizations are needed, such as culturalized training, outreach strategies for the recruitment of underrepresented scholars, and systematic evaluations, to promote diversity, equity, and excellence in public health and psychology research. Without robust financial, institutional, and governmental support, training programs are not sustainable. By integrating these elements, training programs can effectively advance their goals and ensure long-term impact and sustainability.

## Figures and Tables

**Table 1.** Overview of CHANGE program components and their tailored implementation.

Program components	Implementation for CHANGE

Creation of a cohort seminar to hold space for connection, reflection, discussion, and content	CHANGE holds a weekly 2-hour seminar, often with invited speakers from within and beyond UM to present content specific to the program competencies. Sessions also include time to debrief and share reflections on content as well as the application with trainees’ experiences and areas of interest.
Selection of existing coursework aligned with program competencies	Several courses from UM’s Department of Public Health Sciences and Department of Psychology were selected for incorporation into the training plan. Post-doctoral trainees complete 18 credits worth of courses as a part of the ‘Methods to Address Disparities in HIV and Mental Health Research Certificate’.
Promotion of existing internal and external interdisciplinary content	Trainees are encouraged to attend presentations, workshops, and conferences on content aligned with the training program goals and competencies (e.g. CHARMing Conversations led by the Community Advisory Board, Intersectionality Training Institute’s Research Salons, NIH’s Sexual and Gender Minority Health Research Workshop).
Diverse and complimentary mentorship structure	In addition to the three CHANGE Directors, trainees have access to 30+ affiliate mentors chosen for their history of mentorship and/or because of their specific skills, contributions to their fields, and linkages in the community.
Supporting longstanding community partnerships	Trainees are linked to community organizations working with Directors and affiliate mentors based on scope and mutual interests (e.g. Community Partner ‘Speed-Dating’ sessions were held to offer the space for introductory conversations about potential projects).
Leveraging institutional infrastructures supporting early-stage investigators	CHANGE leverages an array of state-of-the-art research training resources, including services and expertise from CHARM’s five cores (e.g. Grant and Manuscript Development support, Peer Reviews, Pilot Funding, IRB consultation, Design and Analysis support, and Consent-to-Contact databases).

*CHARM* – Center for HIV/AIDS Research and Mental Health; *NIH* – National Institutes of Health.

**Table 2.** Trauma-informed facilitation component and implementation description.

Component	Description

Trauma-Informed Facilitation	Training program is set within a safe space with established ‘ground rules’ for discussions and facilitated empathy-building. Speakers from diverse and intersectional backgrounds are intentionally invited.
Co-created CHANGE Seminar Content	Seminar topics are co-created with trainees and Directors and incorporate trauma-informed principles (e.g. safety, trustworthiness transparency, collaboration, mutuality). Trainees participate in seminars aimed at understanding and being responsive to trauma’s impact on mental health and health equity.
Debriefing and Reflective Dialogue	Trainees are encouraged to debrief and reflect on content with peers and Directors, creating a space for processing information and discussing how insights impact their personal and professional growth.
Community-Engaged Research	Trainees learn community-based participatory research strategies and partner with community organizations, which emphasizes equitable partnerships with communities in research projects, aiming for collaborative and culturally sensitive solutions.
Focus Groups for Trainee Feedback	Annual focus groups provide trainees with a structured platform to talk about their experiences, suggest improvements, and discuss needs. The focus group is facilitated by an institutional faculty (non T32 director or mentor) and findings are de-identified before being shared with T32 Directors. Directors use this feedback to enhance trauma-informed training responsiveness.
Examples of Trauma-Focused CHANGE Training Sessions and Invited Speakers	*How -isms drive health disparities* – **Dr. Victoria Behar-Zusman**: Addresses systemic inequities, fostering awareness of interlocking systems of oppression and their impact on health. *Structural Inequities and HIV* – **Dr. Sannisha Dale**: Explores structural inequities to build understanding of trauma-related impacts in research and community work. *Intersectional Approaches to Transgender Health, Reproductive Justice, Health Impacts of State-Sanctioned Violence, and Systemic Racism* – **Dr. Elle Lett**: Covers multifaceted issues relating directly to trauma and systemic oppression. *Black Communities and HIV –* **Dr. Candice Sternberg, Dr. Sonjia Kenya, Jakisha Blackmon, and Dr. Breanne Young**: Focuses on the experiences of Black communities, highlighting racial trauma. *Latinx Communities and HIV* – **Dr. Mariano Kanamori**: Tailors discussions to the Latinx community, emphasizing cultural sensitivity and trauma-informed principles. *Community Assessment and Communication* – **Dr. Audrey Harkness** and **Dr. Victoria Orrego-Dunleavy**: Promotes community engagement through empathy and safe communication strategies, core to trauma-informed facilitation.

## Data Availability

Data sharing is not applicable to this article as no new data were created or analyzed in this study.

## References

[R1] Akintobi TH, Barrett R, Hoffman L, Scott S, Davis K, Jones T, Brown NDV, Fraire M, Fraire R, Garner J, Gruner A, Hill J, Meckel R, Obi C, Omunga P, Parham Q, Rice T, Samples O, & Terrill T. (2023). The community engagement course and action network: Strengthening community and academic research partnerships to advance health equity. Frontiers in Public Health, 11, 11. 10.3389/fpubh.2023.1114868 PMC10317472 37404270

[R2] Archuleta S, Ibrahim H, Pereira TL-B, & Shorey S. (2024). Microaggression interactions among healthcare professionals, trainees and students in the clinical environment: A mixed-studies review. Trauma, Violence & Abuse, 25(5), 3843–3871. 10.1177/15248380241265380 39082181

[R3] Armstrong WS (2021). The human immunodeficiency virus workforce in crisis: An urgent need to build the foundation required to end the epidemic. Clinical Infectious Diseases, 72(9), 1627–1630. 10.1093/cid/ciaa302 32211784

[R4] Bass SB, D’Avanzo P, Alhajji M, Ventriglia N, Trainor A, Maurer L, Eisenberg R, & Martinez O. (2020). Exploring the engagement of racial and ethnic minorities in HIV treatment and vaccine clinical trials: A scoping review of literature and implications for future research. AIDS Patient Care and STDs, 34(9), 399–416. 10.1089/apc.2020.0008 32931317 PMC10722429

[R5] Bath EP, Brown K, Harris C, Guerrero A, Kozman D, Flippen CC, Garraway I, Watson K, Holly L, Godoy SM, Norris K, & Wyatt G. (2022). For us by us: Instituting mentorship models that credit minoritized medical faculty expertise and lived experience. Frontiers in Medicine, 9, 9. 10.3389/fmed.2022.966193 PMC9634999 36341236

[R6] Beames JR, Kikas K, O’Gradey-Lee M, Gale N, Werner-Seidler A, Boydell KM, & Hudson JL (2021). A new normal: Integrating lived experience into scientific data syntheses. Frontiers in Psychiatry, 12, 763005. 10.3389/fpsyt.2021.763005 PMC8585932 34777064

[R7] Boga DJ, & Dale SK (2022). Black women living with HIV: A latent profile analysis of intersectional adversities, resilience, and mental health. AIDS Patient Care and STDs, 36(9), 364–374. 10.1089/apc.2022.0053 36040393 PMC9514596

[R8] Bonifacino E, Ufomata EO, Farkas AH, Turner R, & Corbelli JA (2021). Mentorship of underrepresented physicians and trainees in academic medicine: A systematic review. Journal of General Internal Medicine, 36(4), 1023–1034. 10.1007/s11606-020-06478-7 33532959 PMC7852467

[R9] Bowleg L, Malekzadeh AN, Mbaba M, & Boone CA (2022). Ending the HIV epidemic for all, not just some: Structural racism as a fundamental but overlooked social-structural determinant of the U.S. HIV epidemic. Current Opinion in HIV and AIDS, 17(2), 40–45. 10.1097/COH.0000000000000724 35102051 PMC9109814

[R10] Brandt AM (1978). Racism and research: The case of the tuskegee syphilis study. The Hastings Center Report, 8(6), 21–29. 10.2307/3561468 721302

[R11] Breland-Noble A, Streets FJ, & Jordan A. (2024). Community-based participatory research with black people and black scientists: The power and the promise. Lancet Psychiatry, 11(1), 75–80. 10.1016/S2215-0366(23)00338-3 38101875

[R12] Brown NE, & Montoya C. (2020). Intersectional mentorship: A model for empowerment and transformation. PS, Political Science & Politics, 53(4), 784–787. 10.1017/S1049096520000463

[R13] Brown T, Berman S, McDaniel K, Radford C, Mehta P, Potter J, & Hirsh DA (2021). Trauma-informed medical education (TIME): Advancing curricular content and educational context. Academic Medicine, 96(5), 661. 10.1097/ACM.0000000000003587 32675789

[R14] Brunsma DL, Embrick DG, & Shin JH (2017). Graduate students of color: Race, racism, and mentoring in the white waters of academia. Sociology of Race & Ethnicity, 3(1), 1–13. 10.1177/2332649216681565

[R15] Casanova F, Prasad S, Garolera DB, Riopelle C, & Stoler J. (2023). Hurricane vulnerability and constrained choices among mobile home park residents in South Florida. Weather, Climate, and Society, 15(3), 677–692. 10.1175/WCAS-D-22-0122.1

[R16] Casanova FO, Ramirez E, McKenna M, Seide K, & Cepeda A. (2024). Unveiling intersectional narratives of minority stress among chicano women via a flexible diary study. In The Society for the Study of Social Problems 74th Annual Meeting. https://www.sssp1.org/file/2024AM/Final_Program_Schedule.pdf/

[R17] Cavener J, & Lonbay S. (2024). Enhancing ‘best practice’ in trauma-informed social work education: Insights from a study exploring educator and student experiences. Social Work Education, 43(2), 317–338. 10.1080/02615479.2022.2091128

[R18] Centers for Disease Control and Prevention. (2019). Diagnoses of HIV infection in the United States and dependent areas 2019. Special Focus Profiles. https://www.cdc.gov/hiv/library/reports/hiv-surveillance/vol-32/content/special-focus-profiles.html

[R19] Centers for Disease Control and Prevention. (2023). Expanding PrEP coverage in the United States to achieve EHE goals 2023. https://www.cdc.gov/nchhstp/dear_colleague/2023/dcl-101723-prep-coverage.html

[R20] Cespedes M, Das M, Hojilla JC, Blumenthal J, Mounzer K, Ramgopal M, Hodge T, Torres TS, Peterson C, Shibase S, Elliott A, Demidont AC, Callaghan L, Watson CC, Carter C, Kintu A, Baeten JM, Ogbuagu O, & Newman PA (2022). Proactive strategies to optimize engagement of black, Hispanic/Latinx, transgender, and nonbinary individuals in a trial of a novel agent for HIV pre-exposure prophylaxis (PrEP). PLoS One, 17(6), e0267780. 10.1371/journal.pone.0267780 PMC9165827 35657826

[R21] Coleman JL, Jones M, Washington D, Almirol E, Forberg P, Dyer TV, Spieldenner A, Martinez O, Rodriguez-Diaz CE, Parker SD, Schneider JA, & Brewer R. (2022). Using the meaningful involvement of people living with HIV/AIDS (MIPA) framework to assess the engagement of sexual minority men of color in the US HIV response: A literature review. Journal of Racial and Ethnic Health Disparities, 10(5), 2374–2396. 10.1007/s40615-022-01417-0 36171496 PMC10098811

[R22] Collective CR (1986). The combahee river collective statement: Black feminist organizing in the seventies and eighties. (No Title).

[R23] Collins PH, da Silva ECG, Ergun E, Furseth I, Bond KD, & Martínez-Palacios J. (2021). Intersectionality as critical social theory: Intersectionality as critical social theory, Patricia Hill Collins, duke university press, 2019. Contemporary Political Theory, 20(3), 690. 10.1057/s41296-021-00490-0

[R24] Conron KJ, Luhur W, & Goldberg SK (2021). LGBT adults in large US metropolitan areas. UCLA School of Law Williams Institute.

[R25] Cortes CP, & Sued O. (2024). Reflecting on a decade of progress: Zero discrimination day and the ongoing struggle against transphobia. Journal of the International AIDS Society, 27(3), e26227. 10.1002/jia2.26227 PMC10903327 38421156

[R26] Crenshaw K. (1989). Demarginalizing the intersection of race and sex: A black feminist critique of antidiscrimination doctrine, feminist theory and antiracist politics. 1989, Article 8.(1). https://chicagounbound.uchicago.edu/uclf/vol1989/iss1/8

[R27] Dale SK, Ayala G, Logie CH, & Bowleg L. (2022). Addressing HIV-Related intersectional stigma and discrimination to improve public health outcomes: An AJPH supplement. American Journal of Public Health, 112(S4), S335–S337. 10.2105/AJPH.2022.306738 35763724 PMC9241474

[R28] Dawkins D. (2021). Recruitment and retention of minority high school students to increase diversity in the nursing profession. The Nursing Clinics of North America, 56(3), 427–439. 10.1016/j.cnur.2021.04.007 34366162

[R29] Department of Health and Human Services. (2020). PA-20–142: Ruth L. Kirschstein national research service award (NRSA) institutional research training grant (parent T32). https://grants.nih.gov/grants/guide/pa-files/PA-20-142.html

[R30] Dubé K, Kanazawa J, Campbell C, Boone CA, Maragh-Bass AC, Campbell DM, Agosto-Rosario M, Stockman JK, Diallo DD, Poteat T, Johnson M, Saberi P, & Sauceda JA (2022). Considerations for increasing racial, ethnic, gender, and sexual diversity in HIV cure-related research with analytical treatment interruptions: A qualitative inquiry. AIDS Research & Human Retroviruses, 38(1), 50–63. 10.1089/aid.2021.0023 33947268 PMC8785755

[R31] Edwards KJ, Akam E, Ijoma JN, Mack KN, Pereira PMR, Dhanvantari S, Ta HT, Wang X, Alt K, & Henry KE (2022). Visions by WIMIN: Global mentorship to retain underrepresented trainees. Molecular Imaging and Biology, 24 (4), 519–525. 10.1007/s11307-022-01716-2 35301641 PMC8929712

[R32] Esparza CJ, Simon M, Bath E, & Ko M. (2022). Doing the work—or not: The promise and limitations of diversity, equity, and inclusion in US medical schools and academic medical centers. Equity, and Inclusion in US Medical Schools and Academic Medical Centers Frontiers in Public Health, 10, 10. 10.3389/fpubh.2022.900283 PMC9256912 35812485

[R33] Evans KN, Martinez O, King H, van den Berg JJ, Fields EL, Lanier Y, Hussen SA, Malavé-Rivera SM, Duncan DT, Gaul Z, & Buchacz K. (2023). Utilizing community based participatory research methods in Black/African American and Hispanic/Latinx communities in the US: The CDC minority HIV research initiative (MARI-Round 4). Journal of Community Health, 48(4), 698–710. 10.1007/s10900-023-01209-5 36943607 PMC10028312

[R34] Fernandez SB, Clarke RD, Langwerden RJ, Perez KR, Howard M, Hospital MM, Morris SL, & Wagner EF (2023). Lessons learned from a community–university partnership to increase HIV testing services for emerging adults at a minority-serving institution. Journal of Prevention and Health Promotion, 4(3–4), 339–363. 10.1177/26320770231189182

[R35] Filingeri V, Mendez H, Lin ASY, Burks-Abbott G, Szarkowski A, & Fogler J. (2023). Cultural humility and cultural brokering in professional training: Insights from people of color (POC) and persons with disabilities (PWD). Developmental Disabilities Network Journal, 3(1). 10.59620/2694-1104.1068

[R36] Fitzpatrick LK, Sutton M, & Greenberg AE (2006). Toward eliminating health disparities in HIV/AIDS: The importance of the minority investigator in addressing scientific gaps in black and latino communities. Journal of the National Medical Association, 98(12), 1906–1911. 17225832 PMC2569682

[R37] Florida Department of Health. (2018). Persons living with an HIV diagnosis in Miami-Dade County, 2017. https://miamidade.floridahealth.gov/programs-and-services/infectious-disease-services/hiv-aids-services/_documents/10-26-18-update-HIV-Surveillance/_documents/FS-2017-MIAMI-DADE.pdf

[R38] Florida Department of Health. (2019a). Blacks living with diagnosed HIV infection in Miami-Dade County, 2018. https://miamidade.floridahealth.gov/programs-and-services/infectious-disease-services/hiv-aids-services/_documents/2019/_documents/2018-FS-Blacks.pdf

[R39] Florida Department of Health. (2019b). Hispanics living with diagnosed HIV infection in Miami-Dade County, 2018. https://miamidade.floridahealth.gov/programs-and-services/infectious-disease-services/hiv-aids-services/_documents/2019/_documents/2018-FS-Hispanics-English.pdf

[R40] Florida Department of Health. (2019c). Male-to-Male Sexual Contact [MSM] living with diagnosed HIV infection in Miami-Dade County, 2018. https://miamidade.floridahealth.gov/programs-and-services/infectious-disease-services/hiv-aids-services/_documents/2019/_documents/2018-FS-MSM.pdf

[R41] Forney DJ, Sheehan DM, Dale SK, Li T, De La Rosa M, Spencer EC, & Sanchez M. (2024). The impact of HIV-Related stigma on racial/ethnic disparities in retention in HIV care among adults living with HIV in Florida. Journal of Racial and Ethnic Health Disparities, 11(4), 2498–2508. 10.1007/s40615-023-01715-1 37495905 PMC10811278

[R42] Fu R, Leff SS, Carroll IC, Brizzolara-Dove S, & Campbell K. (2024). Racial microaggressions and anti-racism: A review of the literature with implications for school-based interventions and school psychologists. School Psychology Review, 53(1), 1–16. 10.1080/2372966X.2022.2128601 38487040 PMC10936695

[R43] Fuchs J, Kouyate A, Kroboth L, & McFarland W. (2016). Growing the pipeline of diverse HIV investigators: The impact of mentored research experiences to engage underrepresented minority students. AIDS and Behavior, 20(2), 249–257. 10.1007/s10461-016-1392-z 27066986 PMC4995178

[R44] Gamarel KE, Rebchook G, McCree BM, Jadwin-Cakmak L, Connolly M, Reyes LA, & Sevelius JM (2022). The ethical imperative to reduce HIV stigma through community-engaged, status-neutral interventions designed with and for transgender women of colour in the United States. Journal of the International AIDS Society, 25(S1), e25907. 10.1002/jia2.25907 PMC9274348 35818894

[R45] Gandhi M, & Johnson M. (2016). Creating more effective mentors: Mentoring the mentor. AIDS and Behavior, 20(2), 294–303. 10.1007/s10461-016-1364-3 27039092 PMC4995126

[R46] Garrison HH, & Deschamps AM (2014). NIH research funding and early career physician scientists: Continuing challenges in the 21st century. FASEB Journal, 28(3), 1049–1058. 10.1096/fj.13-241687 24297696 PMC3929670

[R47] Garrison NA (2013). Genomic justice for native americans: Impact of the havasupai case on genetic research. Science, Technology, & Human Values, 38(2), 201–223. 10.1177/0162243912470009 PMC5310710 28216801

[R48] Gilbert L, Goddard-Eckrich D, Chang M, Hunt T, Wu E, Johnson K, Richards S, Goodwin S, Tibbetts R, Metsch LR, & El-Bassel N. (2021). Effectiveness of a culturally tailored HIV and Sexually transmitted infection prevention intervention for black women in community supervision programs: A randomized clinical trial. JAMA Network Open, 4(4), e215226. 10.1001/jamanetworkopen.2021.5226 PMC8035652 33835175

[R49] Greenberg AE, Wutoh A, Bowleg L, Robinson B, Magnus M, Segarra L, Simon P, Wutoh A, Blankenship K, Burke M, Okeke NL, Corneli A, Hussen S, Holliday RC, Ciaranello A, Ghebremichael M, Haberer J, Irvin R, Irvin N, & NIH CFAR Program Office. (2023). Centers for AIDS research (CFAR) diversity, equity, and inclusion pathway initiative (CDEIPI): Developing career pathways for early-stage scholars from racial and ethnic groups underrepresented in HIV science and medicine. Journal of Acquired Immune Deficiency Syndromes, 94(2S), S5–S12. 10.1097/QAI.0000000000003270 37707842 PMC10567097

[R50] Greenwood GL, Wilson A, Bansal GP, Barnhart C, Barr E, Berzon R, Boyce CA, Elwood W, Gamble-George J, Glenshaw M, Henry R, Iida H, Jenkins RA, Lee S, Malekzadeh A, Morris K, Perrin P, Rice E, Sufian M, & Gaist P. (2022). HIV-Related stigma research as a priority at the national institutes of health. HIV-Related Stigma Research as a Priority at the National Institutes of Health AIDS and Behavior, 26(1), 5–26. 10.1007/s10461-021-03260-6 33886010 PMC8060687

[R51] Guilamo-Ramos V, Thimm-Kaiser M, & Benzekri A. (2023). Is the USA on track to end the HIV epidemic? The Lancet HIV, 10(8), e552–e556. 10.1016/S2352-3018(23)00142-X 37541707

[R52] Guilamo-Ramos V, Thimm-Kaiser M, Benzekri A, Chacón G, López OR, Scaccabarrozzi L, & Rios E. (2020). The invisible US Hispanic/Latino HIV crisis: Addressing gaps in the national response. American Journal of Public Health, 110(1), 27–31. 10.2105/AJPH.2019.305309 31725313 PMC6893335

[R53] Gwayi-Chore M-C, Del Villar EL, Fraire LC, Waters C, Andrasik MP, Pfeiffer J, Slyker J, Mello SP, Barnabas R, Moise E, & Heffron R. (2021). “Being a person of color in this institution is exhausting”: Defining and optimizing the learning climate to support diversity, equity, and inclusion at the university of washington school of public health. Equity, and Inclusion at the University of Washington School of Public Health Frontiers in Public Health, 9, 9. 10.3389/fpubh.2021.642477 PMC8082071 33937172

[R54] Hemming J, Eide K, Harwood E, Ali R, Zhu Z, & Cutler J. (2019). Exploring professional development for new investigators underrepresented in the federally funded biomedical research workforce. Ethnicity & Disease, 29(Suppl 1), 123–128. 10.18865/ed.29.S1.123 30906160 PMC6428175

[R55] Ibáñez-Carrasco F, Worthington C, Rourke S, & Hastings C. (2020). Universities without walls: A blended delivery approach to training the next generation of HIV researchers in Canada. International Journal of Environmental Research and Public Health, 17(12), Article 12. 10.3390/ijerph17124265 PMC7344852 32549263

[R56] Irie WC, Chitneni P, Glynn TR, Allen W, Chai PR, Engelman AN, Hurtado R, Li JZ, Li P, Lockman S, Marcus JL, Ogunshola FJ, Rönn MM, Haberer J, Ghebremichael M, Ciaranello A, & For the Harvard University Center for AIDS Research Diversity, E. (2023). Pathways and intersections: Multifaceted approaches to engage individuals from underrepresented and marginalized communities in HIV research and career development. JAIDS Journal of Acquired Immune Deficiency Syndromes, 94(2S), S116. 10.1097/QAI.0000000000003265 37707858 PMC10503030

[R57] Jackson Y, Noll JG, Shenk CE, Connell CM, Lunkenheimer E, & Schreier HMC (2023). The child maltreatment T32 training program at Penn State: Innovation for creating the next generation of scholars in child maltreatment science. In Shenk CE (Ed.), Innovative methods in child maltreatment research and practice: Advances in detection, causal estimation, and intervention (pp. 257–283). Springer International Publishing. 10.1007/978-3-031-33739-0_13

[R58] Jaramillo J, & Harkness A. (2023). Supporting the helpers: What do peer deliverers of HIV interventions need to sustain their implementation efforts? Translational Behavioral Medicine, 13(11), 826–832. 10.1093/tbm/ibad039 37368359 PMC10631879

[R59] Jaramillo J, Reyes N, Atuluru P, Payen N, Taylor K, Safren SA, Saber R, & Harkness A. (2024). Peer ambassador stories: Formative qualitative research to enhance the reach of PrEP, HIV testing, and behavioral health treatments to LMSM in South Florida. AIDS Care, 36(4), 569–579. 10.1080/09540121.2023.2287736 38157344 PMC10932813

[R60] Jenkins C, Bittner Fagan H, Passarella J, Fournakis N, & Burshell D. (2020). Training academic and community Investigator teams for community-engaged research: Program development, implementation, evaluation and replication. Progress in Community Health Partnerships: Research, Education, and Action, 14(2), 229–242. 10.1353/cpr.2020.0019 33416644 PMC8392131

[R61] Jenkins S. (2022). Wading in white waters: Black non-tenure-track faculty’s lived experiences with racial microaggressions in higher education and their recommendations for institutions, departments and DEI administrators. Education doctoral. https://fisherpub.sjf.edu/education_etd/546

[R62] Jones NK, Ikpeze A, Arena J, & Oyeku SO (2022). Being the first only different: Generational perspectives in driving institutional change in academia. In Laraque-Arena D, Germain L, Young V, & Laraque-Ho R. (Eds.), Leadership at the intersection of gender and race in healthcare and science (pp. 32–48). Routledge.

[R63] Julian McFarlane S, Occa A, Peng W, Awonuga O, & Morgan SE (2022). Community-based participatory research (CBPR) to enhance participation of racial/ethnic minorities in clinical trials: A 10-year systematic review. Health Communication, 37(9), 1075–1092. 10.1080/10410236.2021.1943978 34420460

[R64] Koethe JR, Eeds A, Stewart LV, Haas DW, Hildreth JEK, Mallal S, Wanjalla C, Perkins J, Ahonkhai A, Dong X, Berhanu R, & Dash C. (2023). The tennessee center for AIDS research HIV research training program for minority high school and undergraduate students: Development, implementation, and early outcomes. Journal of Acquired Immune Deficiency Syndromes, 94(2S), S42–S46. 10.1097/QAI.0000000000003261 37707847 PMC10503046

[R65] Kreider EF, Ortega-Burgos Y, Dumeng-Rodriguez J, Gesualdi J, O’Brien C, Bracy D, Johnson J, Bowman J, Metzger D, Dine CJ, Favor K, Jordan-Sciutto KL, & Momplaisir F. (2023). Early engagement in HIV research: Evaluation of the penn CFAR scholars program aimed at increasing diversity of the HIV/AIDS workforce. Journal of Acquired Immune Deficiency Syndromes, 94(2S), S28–S35. 10.1097/QAI.0000000000003260 37707845 PMC10754256

[R66] Kross EK, Rosenberg AR, Engelberg RA, & Curtis JR (2020). Postdoctoral research training in palliative care: Lessons learned from a T32 program. Journal of Pain and Symptom Management, 59(3), 750–760.e8. 10.1016/j.jpainsymman.2019.11.013 31775020 PMC7029795

[R67] Li DH, Macapagal K, Mongrella M, Saber R, & Mustanski B. (2024). “Your package could not Be delivered”: The state of digital HIV intervention implementation in the US. Current HIV/AIDS Reports, 21(3), 152–167. 10.1007/s11904-024-00693-1 38502421 PMC11710848

[R68] Londono E, Casanova FO, Ashad Bishop K, & Ashad Bishop K. (2024). Community experiences with COVID-19 eviction moratoriums in Florida. In The Society for the Study of Social Problems 74th Annual Meeting. https://www.sssp1.org/file/2024AM/Final_Program_Schedule.pdf1

[R69] Magnus M, Segarra L, Robinson B, Blankenship K, Corneli A, Ghebremichael M, Irvin N, McIntosh R, Favor KE, Jordan-Sciutto KL, Kimberly J, Sluis-Cremer N, Koethe JR, Newell A, Wood C, Rana A, Stockman JK, Sauceda J, Marquez C, & Greenberg AE (2023). Impact of a multi-institutional initiative to engage students and early-stage scholars from underrepresented racial and ethnic minority groups in HIV research: The centers for AIDS research diversity, equity, and inclusion pathway initiative. Journal of Acquired Immune Deficiency Syndromes, 94(2S), S13–S20. 10.1097/QAI.0000000000003266 37707843 PMC10539009

[R70] Marshall A, & Cahill S. (2022). Barriers and opportunities for the mental health of LGBT older adults and older people living with HIV: A systematic literature review. Aging & Mental Health, 26(9), 1845–1854. 10.1080/13607863.2021.2003300 34784488

[R71] Martinez-Cola M. (2020). Collectors, nightlights, and allies, oh my. Understanding & Dismantling Privilege, 10(1), 61–82.

[R72] Mehrabadi A, Austin N, Keyes KM, & De Vera MA (2024). It’s personal: Navigating research questions that stem from our lived experiences. International Journal of Epidemiology, 53(6), dyae132. 10.1093/ije/dyae132 PMC11471260 39396252

[R73] Meyers FJ, Mathur A, Fuhrmann CN, O’Brien TC, Wefes I, Labosky PA, Duncan DS, August A, Feig A, Gould KL, Friedlander MJ, Schaffer CB, Van Wart A, & Chalkley R. (2016). The origin and implementation of the broadening experiences in scientific training programs: An NIH common fund initiative. FASEB Journal, 30(2), 507–514. 10.1096/fj.15-276139 26432783 PMC6188226

[R74] Morgan J, Schwartz C, Ferlatte O, Mniszak C, Lachowsky N, Jollimore J, Hull M, & Knight R. (2021). Community-based participatory approaches to knowledge translation: HIV prevention case study of the investigaytors program. Archives of Sexual Behavior, 50(1), 105–117. 10.1007/s10508-020-01789-6 32737658

[R75] Morrow SL (2005). Quality and trustworthiness in qualitative research in counseling psychology. Journal of Counseling Psychology, 52(2), 250. 10.1037/0022-0167.52.2.250

[R76] Muhammad M, Wallerstein N, Sussman AL, Avila M, Belone L, & Duran B. (2015). Reflections on Researcher identity and power: The impact of positionality on community based participatory research (CBPR) processes and outcomes. Critical Sociology, 41(7–8), 1045–1063. 10.1177/0896920513516025 27429512 PMC4943756

[R77] National Institutes of Health. (2024). Kirschstein-nrsa institutional research training grants (T32s): Competing applications, awards, and success rates. https://www.report.nih.gov/nihdatabook/report/60

[R78] Nearing KA, Nuechterlein BM, Tan S, Zerzan JT, Libby AM, & Austin GL (2020). Training mentor–mentee pairs to build a robust culture for mentorship and a pipeline of clinical and translational researchers: The colorado mentoring training program. Academic Medicine, 95(5), 730. 10.1097/ACM.0000000000003152 31972672 PMC7644265

[R79] Nguyen M, Nguyen ND, Chaudhry SI, Desai MM, Cavazos JE, & Boatright D. (2022). Inequity in national institutes of health predoctoral fellowships, 2001–2020. Inequity in National Institutes of Health Predoctoral Fellowships, 2001–2020 JAMA Network Open, 5(10), 5(10). 10.1001/jamanetworkopen.2022.38600 PMC9606849 36287568

[R80] NIH RePORTER. (2013). Biopsychosocial research training in immunology and AIDS. https://reporter.nih.gov/search/ejxnlkZ09EWNO3wgEtvELA/project-details/8470486

[R81] NIH RePORTER. (2024a). Addressing disaster displacement for recovery with equitable and sustainable systems (ADDRESS) of HIV care in Puerto rico. https://reporter.nih.gov/search/suSzzwqm_kqTLfjnheupWA/project-details/11085526

[R82] NIH RePORTER. (2024b). Employment as prevention: Adapting a structural intervention to achieve HIV equity among immigrant latino MSM. https://reporter.nih.gov/search/X_wAU6482U6hjYBP1pKIWQ/projects

[R83] Nosyk B, Zang X, Krebs E, Enns B, Min JE, Behrends CN, Del Rio C, Dombrowski JC, Feaster DJ, Golden M, Marshall BDL, Mehta SH, Metsch LR, Pandya A, Schackman BR, Shoptaw S, Strathdee SA, Behrends CN, Del Rio C, & Strathdee SA (2020). Ending the HIV epidemic in the USA: An economic modelling study in six cities. The Lancet HIV, 7(7), e491–e503. 10.1016/S2352-3018(20)30033-3 32145760 PMC7338235

[R84] Nowotny KM, Zielinski MJ, Stringer KL, Pugh T, Wu E, Metsch LR, El-Bassel N, Nunn AS, & Beckwith CG (2020). Training the next generation of researchers dedicated to improving health outcomes for justice-involved populations. American Journal of Public Health, 110(S1), S18–S20. 10.2105/AJPH.2019.305411 31967875 PMC6987935

[R85] Park SK, Chen AMH, Daugherty KK, Frankart LM, & Koenig RA (2023). A scoping review of the hidden curriculum in pharmacy education. American Journal of Pharmaceutical Education, 87(3), ajpe8999. 10.5688/ajpe8999 PMC10159550 36220178

[R86] Patel D, Clark HA, Williams WO, Taylor-Aidoo N, & Wright C. (2024). CDC-Funded HIV testing services outcomes and social determinants of health in ending the HIV epidemic in the U.S. Jurisdictions. AIDS and Behavior, 28(4), 1152–1165. 10.1007/s10461-023-04133-w 37479920 PMC10799961

[R87] Patel ZS, Brodar KE, Hylton E, Glynn TR, & Dale SK (2023). Integrating public health core values into psychology training competencies. Training and Education in Professional Psychology, 17(3), 248–258. 10.1037/tep0000419

[R88] Pellowski JA, Kalichman SC, Matthews KA, & Adler N. (2013). A pandemic of the poor: Social disadvantage and the U.S. HIV epidemic. The American Psychologist, 68(4), 197–209. 10.1037/a0032694 23688088 PMC3700367

[R89] Peña M-M, Bonachea E, Bell M, Duara J, Okito O, Barrero-Castillero A, & Anani UE (2023). Recommendations to improve recruitment and retention of underrepresented in medicine trainees in neonatal-perinatal medicine. Journal of Perinatology, 43(4), Article 4. 540–545. 10.1038/s41372-022-01552-w 36329162

[R90] Pfund C, Sancheznieto F, Byars-Winston A, Zárate S, Black S, Birren B, Rogers J, & Asai DJ (2022). Evaluation of a culturally responsive mentorship education program for the advisers of howard hughes medical institute gilliam program graduate students. CBE - Life Sciences Education, 21(3), ar50. 10.1187/cbe.21-11-0321 PMC9582832 35862583

[R91] Philbin MM, Guta A, Wurtz H, Kinnard EN, Bradley-Perrin I, & Goldsamt L. (2022). How black and latino young men who have sex with men in the United States experience and engage with eligibility criteria and recruitment practices: Implications for the sustainability of community-based research. Critical Public Health, 32(5), 677–688. 10.1080/09581596.2021.1918329 36439240 PMC9697991

[R92] Phillips G, McCuskey D, Ruprecht MM, Curry CW, & Felt D. (2021). Structural interventions for HIV prevention and care among US men who have sex with men: A systematic review of evidence, gaps, and future priorities. AIDS and Behavior, 25(9), 2907–2919. 10.1007/s10461-021-03167-2 33534056 PMC7856612

[R93] Pitpitan EV, Campbell CK, Zúñiga ML, Strathdee SA, & Stockman JK (2023). Supporting and uplifting new and diverse scientists in HIV research (San Diego SUN): A research education and training program to promote the success of black, indigenous, and people of color predoctoral and postdoctoral fellows. Journal of Acquired Immune Deficiency Syndromes, 94(2S), S36–S41. 10.1097/QAI.0000000000003251 37707846 PMC10503063

[R94] Pohlhaus JR, Jiang H, Wagner RM, Schaffer WT, & Pinn VW (2011). Sex differences in application, success, and funding rates for NIH extramural programs. Academic Medicine, 86(6), 759–767. 10.1097/ACM.0b013e31821836ff 21512358 PMC3379556

[R95] Ramasubramanian S, Riewestahl E, & Landmark S. (2021). The trauma-informed equity-minded asset-based model (TEAM): The six r’s for social justice-oriented educators. https://oaktrust.library.tamu.edu/handle/1969.1/194130

[R96] Robillard AG, Julious CH, Smallwood SW, Douglas M, Gaddist BW, & Singleton T. (2022). Structural inequities, HIV community-based organizations, and the end of the HIV epidemic. American Journal of Public Health, 112(3), 417–425. 10.2105/AJPH.2021.306688 35196039 PMC8887177

[R97] Rodriguez-Diaz CE, Martinez O, Bland S, & Crowley JS (2021). Ending the HIV epidemic in US latinx sexual and gender minorities. Lancet, 397(10279), 1043–1045. 10.1016/S0140-6736(20)32521-6 33617767 PMC8684813

[R98] Samuels E, Janevic MR, Harper AE, Lyden AK, Jay GM, Champagne E, & Murphy SL (2024). Updating and evaluating a research best practices training course for social and behavioral research professionals. Journal of Clinical and Translational Science, 8(1), e12. 10.1017/cts.2023.702 38384926 PMC10877512

[R99] Sancheznieto F, Sorkness CA, Attia J, Buettner K, Edelman D, Hobbs S, McIntosh S, McManus LM, Sandberg K, Schnaper HW, Scholl L, Umans JG, Weavers K, Windebank A, & McCormack WT (2022). Clinical and translational science award T32/TL1 training programs: Program goals and mentorship practices. Journal of Clinical and Translational Science, 6(1), e13. 10.1017/cts.2021.884 35211339 PMC8826009

[R100] Schmidt RD, Horigian VE, Duan R, Traynor ST, Davis CA, Gonzalez ST, Forney DJ, Mandler R, Del Rio C, Metsch LR, & Feaster DJ (2024). Psychosocial factors linked to uncontrolled infection and mortality among people living with HIV who use substances: A latent class analysis. AIDS and Behavior, 28(11), 3748–3757. 10.1007/s10461-024-04410-2 39093354 PMC11471706

[R101] Settles IH, Jones MK, Buchanan NT, & Dotson K. (2021). Epistemic exclusion: Scholar(ly) devaluation that marginalizes faculty of color. Journal of Diversity in Higher Education, 14(4), 493–507. 10.1037/dhe0000174

[R102] Shahid NN, & Dale SK (2024). Gendered racial microaggressions, self-silencing, substance use, and HIV outcomes among black women living with HIV: A structural equation modeling approach. AIDS and Behavior, 28(4), 1276–1290. 10.1007/s10461-023-04157-2 37642823 PMC11505459

[R103] Singh U, Levy J, Armstrong W, Bedimo R, Creech CB, Lautenbach E, Popovich KJ, Snowden J, Vyas JM, & Infectious Diseases Society of America, H. M. A. and Pediatric Infectious Diseases Society. (2018). Policy recommendations for optimizing the infectious diseases physician-scientist workforce. The Journal of Infectious Diseases, 218 (suppl_1), S49–S54. 10.1093/infdis/jiy246 30124981

[R104] Steinbach WJ, Benjamin DK, & Sleasman JW (2018). Funding pediatric subspecialty training: Are T32 grants the future? The Journal of Pediatrics, 202, 4–7.e1. 10.1016/j.jpeds.2018.08.035 30360878

[R105] Stoff DM, Bowleg L, Del Río-González AM, Rodriguez-Diaz CE, & Zea MC (2023). Critical perspectives on expanding Racial/Ethnic diversity in the HIV research workforce: Comorbidities and mentoring. Health Education & Behavior, 50(6), 748–757. 10.1177/10901981231157795 PMC10977082 36924258

[R106] Sutton MY, Martinez O, Brawner BM, Prado G, Camacho-Gonzalez A, Estrada Y, Payne-Foster P, Rodriguez-Diaz CE, Hussen SA, Lanier Y, van den Berg JJ, Malavé-Rivera SM, Hickson DA, & Fields EL (2021). Vital voices: HIV prevention and care interventions developed for disproportionately affected communities by historically underrepresented, early-career scientists. Journal of Racial and Ethnic Health Disparities, 8(6), 1456–1466. 10.1007/s40615-020-00908-2 33128188 PMC7598237

[R107] Torres Acosta MA, Chandra S, Li S, Yoon E, Selgrade D, Quinn J, & Ardehali H. (2023). The impact of underrepresented minority or marginalized identity status on training outcomes of MD-PhD students. BMC Medical Education, 23(1), 428. 10.1186/s12909-023-04399-7 37291579 PMC10251672

[R108] U.S. Census Bureau. (2022). Miami-Dade County, Florida, 2022 population estimates. https://www.census.gov/quickfacts/fact/table/miamidadecountyflorida,FL/PST045223

[R109] Valantine HA, Lund PK, & Gammie AE (2016). From the NIH: A systems approach to increasing the diversity of the biomedical research workforce. CBE Life Sciences Education, 15(3), fe4. 10.1187/cbe.16-03-0138 PMC5008902 27587850

[R110] Veale JF, Deutsch MB, Devor AH, Kuper LE, Motmans J, Radix AE, & St. Amand C. (2022). Setting a research agenda in trans health: An expert assessment of priorities and issues by trans and nonbinary researchers. International Journal of Transgender Health, 23(4), 392–408. 10.1080/26895269.2022.2044425 36324879 PMC9621229

[R111] Vitsupakorn S, Pierce N, & Ritchwood TD (2023). Cultural interventions addressing disparities in the HIV prevention and treatment cascade among Black/African americans: A scoping review. BMC Public Health, 23(1), 1748. 10.1186/s12889-023-16658-9 37679765 PMC10485990

[R112] Wallerstein N, Oetzel JG, Sanchez-Youngman S, Boursaw B, Dickson E, Kastelic S, Koegel P, Lucero JE, Magarati M, Ortiz K, Parker M, Peña J, Richmond A, & Duran B. (2020). Engage for equity: A long-term study of community-based participatory research and community-engaged research practices and outcomes. Health Education & Behavior, 47(3), 380–390. 10.1177/1090198119897075 32437293 PMC8093095

[R113] Watson CC, Wilton L, Lucas JP, Bryant L, Victorianne GD, Aradhya K, Fields SD, Wheeler DP, & on behalf of the HPTN Black Caucus. (2020). Development of a black caucus within the HIV prevention trials network (HPTN): Representing the perspectives of black men Who have Sex with men (MSM). International Journal of Environmental Research and Public Health, 17(3), Article 3. 10.3390/ijerph17030871 PMC7037695 32028553

[R114] Webb J, Arthur R, McFarlane-Edmond P, Burns T, & Warren D. (2022a). An evaluation of the experiences of the hidden curriculum of black and minority ethnic undergraduate health and social care students at a London university. Journal of Further and Higher Education, 46(3), 312–326. 10.1080/0309877X.2021.1915967

[R115] Webb TJ, Guerau de Arellano M, Jones HP, Butts CL, Sanchez-Perez L, & Montaner LJ (2022). The minority scientists’ experience: Challenging and overcoming barriers to enhancing diversity and career advancement. The Journal of Immunology, 208(2), 197–202. 10.4049/jimmunol.2101077 35017208 PMC9206815

[R116] Widge AS, Jordan A, Kraguljac NV, Sullivan CRP, Wilson S, Benton TD, Alpert JE, Carpenter LL, Krystal JH, Nemeroff CB, & Dzirasa K. (2023). Structural racism in psychiatric research careers: Eradicating barriers to a more diverse workforce. The American Journal of Psychiatry, 180(9), 645–659. 10.1176/appi.ajp.20220685 37073513 PMC11227892

[R117] Winkelmes M-A (2023). Introduction to transparency in learning and teaching. Perspectives in Learning, 20(1). https://csuepress.columbusstate.edu/pil/vol20/iss1/2

[R118] Wright AJ, Williams NJ, Starling T, Reynolds A, & Garcia-Lavin B. (2023). Deep-structure curriculum liberation for social responsiveness in graduate health service psychology training. Training and Education in Professional Psychology, 17(1), 22–30. 10.1037/tep0000434

[R119] Wright IA, Reid R, Shahid N, Ponce A, Nelson CM, Sanders J, Gardner N, Liu J, Simmons E, Phillips A, Pan Y, Alcaide ML, Rodriguez A, Ironson G, Feaster DJ, Safren SA, & Dale SK (2022). Neighborhood characteristics, intersectional discrimination, mental health, and HIV outcomes among Black women living with HIV, Southeastern United States, 2019‒2020. American Journal of Public Health, 112(S4), S433–S443. 10.2105/AJPH.2021.306675 35763751 PMC9241469

[R120] Zambrana RE, Ray R, Espino MM, Castro C, Douthirt Cohen B, & Eliason J. (2015). “Don’t leave Us behind”: The importance of mentoring for underrepresented minority faculty. American Educational Research Journal, 52(1), 40–72. 10.3102/0002831214563063

[R121] Zang X, Piske M, Humphrey L, Enns B, Sui Y, Marshall BDL, Goedel WC, Feaster DJ, Metsch LR, Sullivan PS, Tookes HE, & Nosyk B. (2023). Estimating the epidemiological impact of reaching the objectives of the Florida integrated HIV prevention and care plan in Miami-Dade County. The Lancet Regional Health - Americas, 27, 27. 10.1016/j.lana.2023.100623 PMC10624567 37928440

[R122] Zhou B, & Louie AK (2022). Beyond humility: Empowering minoritized learners through culturally reflective medicine. Academic Medicine, 97(9), 1299–1304. 10.1097/ACM.0000000000004744 35583951

